# Genetic insights into superior grain number traits: a QTL analysis of wheat-*Agropyron cristatum* derivative pubing3228

**DOI:** 10.1186/s12870-024-04913-z

**Published:** 2024-04-11

**Authors:** Jiansheng Wang, Erwei Wang, Shiping Cheng, Aichu Ma

**Affiliations:** 1 College of Chemistry and Environment Engineering, Pingdingshan University, North to Weilailu road, New district, Pingdingshan, Henan 467000 China; 2 Henan Key Laboratory of Germplasm Innovation and Utilization of Eco-economic Woody Plant, Pingdingshan, Henan China; 3 Pingdingshan Academy of Agricultural Science, Pingdingshan, Henan 467001 China

**Keywords:** Grain number per Spike, Pubing3228, Quantitative trait loci, Candidate gene, Genetic analysis, Wheat breeding

## Abstract

**Background:**

*Agropyron cristatum* (L.) is a valuable genetic resource for expanding the genetic diversity of common wheat. Pubing3228, a novel wheat-*A. cristatum* hybrid germplasm, exhibits several desirable agricultural traits, including high grain number per spike (GNS). Understanding the genetic architecture of GNS in Pubing3228 is crucial for enhancing wheat yield. This study aims to analyze the specific genetic regions and alleles associated with high GNS in Pubing3228.

**Methods:**

The study employed a recombination inbred line (RIL) population derived from a cross between Pubing3228 and Jing4839 to investigate the genetic regions and alleles linked to high GNS. Quantitative Trait Loci (QTL) analysis and candidate gene investigation were utilized to explore these traits.

**Results:**

A total of 40 QTLs associated with GNS were identified across 16 chromosomes, accounting for 4.25–17.17% of the total phenotypic variation. Five QTLs (Q*Gns.wa-1D*, Q*Gns.wa-5 A*, Q*Gns.wa-7Da.1*, Q*Gns.wa-7Da.2* and Q*Gns.wa-7Da.3*) accounter for over 10% of the phenotypic variation in at least two environments. Furthermore, 94.67% of the GNS QTL with positive effects originated from Pubing3228. Candidate gene analysis of stable QTLs identified 11 candidate genes for GNS, including a senescence-associated protein gene (*TraesCS7D01G148000*) linked to the most significant SNP (AX-108,748,734) on chromosome 7D, potentially involved in reallocating nutrients from senescing tissues to developing seeds.

**Conclusion:**

This study provides new insights into the genetic mechanisms underlying high GNS in Pubing3228, offering valuable resources for marker-assisted selection in wheat breeding to enhance yield.

**Supplementary Information:**

The online version contains supplementary material available at 10.1186/s12870-024-04913-z.

## Introduction

Wheat (*Triticum aestivum* L.) is an essential cereal crop globally [1]. While there have been significant increases in wheat yield and total production in recent decades, the current level of wheat grain production is insufficient to meet the future demands of a growing global population [2]. Moreover, this challenge is exacerbated by the gradual reduction of arable land [3, 4]. Thus, improving wheat grain yield is vital for global food and nutrition security [5]. Consequently, increasing grain yield has become the primary goal in wheat breeding.

The yield of wheat is primarily determined by three components: grain number per spike (GNS), grain weight, and spike number [6, 7]. Previous studies have shown that GNS significantly impacts wheat grain yield more than grain weight [8–10]. In wheat breeding programs, high yield is primarily achieved by increasing GNS rather than grain weight [11–15]. Exploring the genetic variation of GNS holds excellent potential for future yield improvement [16]. GNS is a complex quantitative trait controlled by multiple genes [17–19]. Quantitative trait locus (QTL) mapping effectively analyzes such traits [17, 20, 21]. With the development of molecular markers, numerous QTL analyses of GNS have been conducted, leading to the identification of several QTLs [22–24]. These reports have identified QTLs distributed across the entire wheat genome.

Saturated genetic linkage maps play a crucial role in QTL mapping by providing measurements of marker effects and offering useful DNA markers for marker-assisted selection (MAS) in breeding practices [25, 26]. However, due to limited molecular markers, the use of unsaturated genetic linkage maps in the past has led to the presence of linked markers that are further from the target QTL genetic distance [27–29]. This condition has restricted the understanding of the genetic architecture of GNS and the application of markers in wheat breeding [30]. With the development of next-generation sequencing (NGS) technologies, reference genomes for Chinese Spring wheat and other varieties have been published, providing a way to overcome these limitations [31]. NGS-based SNP genotyping platforms have recently been developed, leading to significant progress in QTL analysis for wheat GNS [32, 33]. High-density SNP genotyping arrays, such as the 9 K, 55 K, 660 K, and 820 K SNP arrays, have been developed for wheat and widely used for QTL analysis [34, 35]. For instance, the 90 K wheat SNP chip was employed to study the genetic control of yield-related traits in 66 elite wheat varieties derived from Xiaoyan 6, resulting in the identification of 803 significant marker-trait associations that explained up to 35.0% of the phenotypic variation.

To address the limited genetic diversity in common wheat, it is crucial to identify new genetic loci controlling yield-related traits to broaden genetic variation and accelerate wheat breeding improvements [36, 37]. Wild relatives, such as the *Agropyron* genus, are necessary gene resources for improving common wheat [38, 39]. Intergeneric hybrids between common wheat and *Agropyron cristatum* (*A. cristatum*) have been generated successfully to transfer beneficial allele genes from the *Agropyron* genus to wheat [40, 41]. Several novel wheat-*A cristatum* resources with desirable agronomic traits have been produced [42, 43]. Furthermore, studies have been conducted on the genetic mechanisms of *A. cristatum* in the background of common wheat, focusing on chromosomal segments or genes [44, 45]. For example, a previous study has studied Pubing2978, a wheat-*A.* Cristate translocation line with high GNS using techniques like genomic in situ hybridization (GISH), fluorescence in situ hybridization (FISH), and molecular markers. The *A. cristatum* 6P chromosomal segment is critical in increasing GNS [45]. Another study has identified an enhancer grain weight locus on the 7P chromosome of *A. cristatum* and mapped it to 7PS1-2 using 158 STS markers. Further studies on the mechanism of this locus on wheat grain weight revealed that two translocation lines with 7P chromosomal segments (7PT-A18 and 7PT-B4) could simultaneously increase grain weight, length, and width [41].

Previous studies have mainly focused on common wheat when investigating the genetic factors influencing GNS and have identified several QTLs. However, there is still limited knowledge about the molecular mechanisms controlling GNS in wheat germplasm derived from crossing common wheat with *A. cristatum*, such as the newly bred wheat germplasm, Pubing3228 [46]. To address this gap, we developed a population of RILs derived from crossing Pubing3228 with Jing4839, which exhibits significant variations in GNS. Based on the recently developed wheat 55 K gene genotyping array, this study aimed to identify novel genetic regions and favorable alleles associated with GNS.

Therefore, the integration of advanced genomic technologies and innovative breeding approaches is essential for fulfilling the increasing demand for wheat. By integrating environmental and climatic considerations into breeding efforts, we can deepen our understanding of their influence on GNS and other critical yield determinants. Analyzing the relationships among GNS, grain weight, and spike number across varied environmental settings offers key insights for harmonizing yield component enhancement. Exploring the genetic diversity of common wheat and its wild relatives, especially untapped genetic resources, opens novel pathways for breeding initiatives. The advent of high-throughput phenotyping and sophisticated bioinformatics tools accelerates identifying and applying novel QTLs and genetic markers. Additionally, elucidating the role of *A. cristatum-*related genes in the wheat genome may pave new avenues for enhancing the resilience of wheat to stress and adaptability to various agricultural conditions.

## Materials and methods

### QTL analysis method for novel wheat germplasm Pubing3228 RIL population

For the QTL analysis, this study utilized a RIL population consisting of 210 families derived from the cross between Pubing3228 and Jing4839. Pubing3228, a genetically stable derivative breed, was selected from the offspring of the wheat-*A. cristatum* chromosome addition line 4844-12 (2n = 44). This hybrid germplasm, developed by Professor Li Lihui at the Chinese Academy of Agricultural Sciences over several decades, possesses elite wheat germplasm traits, including long spikes, a large number of spikelets per spike, and a high GNS [46, 47]. Conversely, Jing4839 is characterized by a higher grain weight but a lower GNS. Significant differences in several yield traits, especially in GNS, were observed between the two parents. The F_9_ RIL population was generated through successive selfing of the F_1_ generation derived from crossing Pubing3228 and Jing4839, employing the single seed descent method.

### Collection of phenotypic data for interregional field trials and novel wheat germplasm Pubing3228 progeny

Field experiments were carried out at three locations, namely Pingdingshan, Yangling, and Xianyang, using 210 RILs derived from the cross between Pubing3228 and Jing4839, along with their respective parents. The experiments were conducted over three crop years (2018, 2019, 2020) in Pingdingshan, two crop years (2019 and 2020) in Yangling, and one crop year (2020) in Xianyang. The field layout was organized in a randomized complete block design, with each design replicated three times. Each replication consisted of three rows of crops, each row being 2 m in length and spaced 30 cm apart. Field management was conducted in accordance with local standards. Ten plants from each replication were selected for harvesting and manual threshing upon reaching physiological maturity. The GNS was counted directly, and the data focused on recording the average values related to the central spike of every ten plants.

Field management practices were governed by local standards. When physiological maturity was reached, ten individuals were randomly selected from each replication for harvesting and subsequent manual threshing. The spikes obtained from this process were directly counted to ascertain the GNS, with the data pertaining to the central spike of each plant being systematically recorded.

### DNA extraction and SNP genotyping of hybrid offspring in wheat

Genomic DNA was extracted from the RIL population and its progenitors employing the sodium dodecyl sulfate (SDS) method [48]. Agarose gel electrophoresis was conducted to verify DNA quality using a 0.8% gel. The concentration of DNA was quantified utilizing a microplate reader to ensure compliance with the criteria for further analyses. Subsequently, the DNA samples underwent genotyping at Beijing Capital Bio Company, utilizing the Illumina Infinium iSelect 55 K SNP array. During data processing, specific markers were selectively excluded to enhance the genotyping data’s accuracy and dependability. Notably, markers with a minimum allele frequency (MAF) under 5%, those exhibiting more than 10% missing data, and markers with heterozygosity rates above 20% were removed. Following this filtration process, 3334 high-quality single nucleotide polymorphism (SNP) markers were retained for further QTL analysis. These markers were employed to explore the presence and effect of genetic variants on the targeted traits.

### Construction and localization of QTL linkage map in hybrid offspring of wheat

In this investigation, outliers within the phenotypic data were initially removed before calculating the best linear unbiased estimates (BLUEs) based on the average values for each environment, which then facilitated subsequent QTL mapping analysis. The QTL mapping analysis employed the QTL IciMapping software V4.2 (http://www.isbreeding.net) to construct a genetic linkage map using polymorphic SNP markers between Pubing3228 and Jing4839. The initial step involved the application of the BIN function to the SNP markers, facilitating the identification of segregating distortions, missing data, and superfluous markers. Following this preliminary screening, the residual SNP markers were employed to establish the framework of the genetic linkage map via the MAP function. The Kosambi function and maximum likelihood estimation were applied to ascertain the order and distance of the markers.

Subsequently, the generated genetic linkage map, in conjunction with the IciMapping software V4.2, was applied to execute QTL mapping. The composite interval mapping (ICIM) model was selected to identify QTLs associated with Grain Number per Spike (GNS). A QTL was deemed significant if it exhibited a Log of Odds (LOD) score exceeding 2.5. The QTL analysis was performed individually for each environment, and QTLs identified in two or more environments were regarded as stable. The nomenclature for the wheat QTL adhered to the following pattern: Q*Gns.wa-1D*, where “Q” signifies QTL, succeeded by the trait indicator; “*wa*” denotes the laboratory, and “*1D*” indicates the chromosome.

### Identification of candidate genes associated with GNS in wheat

The selection of candidate genes for GNS was not limited to sequences associated with grain traits. Instead, genes located within the physical intervals of QTL and expressed explicitly in grain tissues were identified as potential candidates. Information regarding these candidate genes was sourced from the JBrowse website (https://urgi.versailles.inra.fr/jbrowseiwgsc). Expression profiles of the candidate genes across various tissues were obtained from the expVIP website (http://wheat-expression.com). By comparing the expression patterns of these preliminary candidate genes, those associated with GNS were determined. This approach facilitated precise genomic localization of genes potentially influencing GNS and further validated their functional relevance through expression profile analysis.

## Results

### Environmental response of wheat varieties Pubing3228 and Jing4839 to GNS and their contributions to the analysis of QTL

**Fig. 1:**
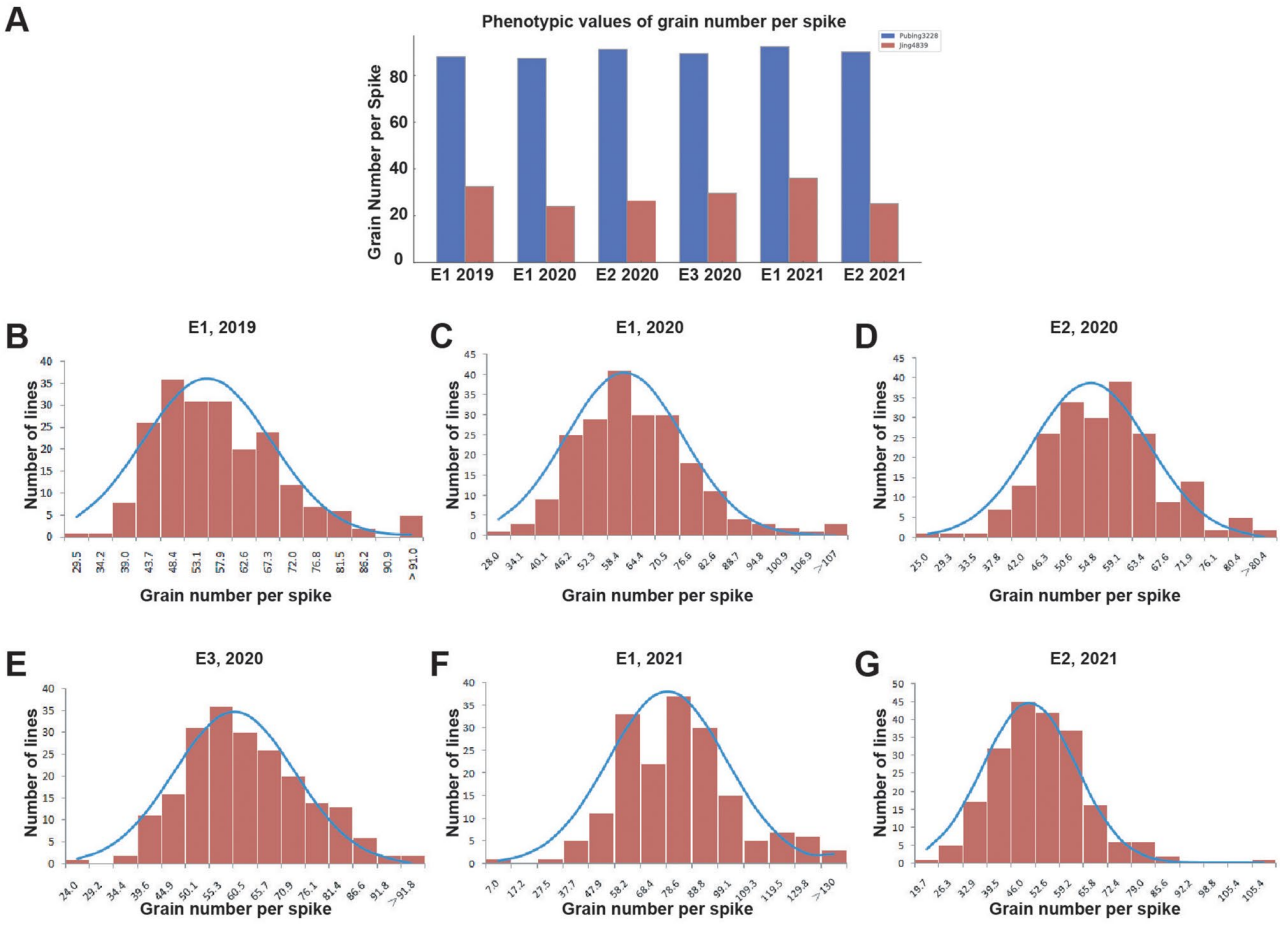
Distribution of GNS in the Pubing3228 and Jing4839 RIL population across different years and locations. *Note* (**A**) Comparative bar chart of GNS from 2019 to 2021, where E1, E2, and E3 represent the distinct environments of Pingdingshan, Yangling, and Xianyang, respectively. Blue bars indicate the Pubing3228 variety, while red bars represent the Jing4839 variety. (**B**-**G**) Histograms and fitted curves showing the distribution of GNS in the RIL population under the environments of Pingdingshan in 2019 (E1, 2019), Pingdingshan in 2020 (E1, 2020), Yangling in 2020 (E2, 2020), Xianyang in 2020 (E3, 2020), Pingdingshan in 2021 (E1, 2021), and Yangling in 2021 (E2, 2021)

This study focused on comparing the performance of GNS in two wheat varieties, Pubing3228 and Jing4839, under identical environmental conditions. The results revealed significant differences in GNS between Pubing3228 and Jing4839, with Pubing3228 consistently exhibiting higher GNS than Jing4839 across all tested environments (Table 1; Fig. 1A). To further investigate the genetic control of GNS, a RIL population derived from the hybridization of these two parental varieties was analyzed for GNS variability across three environments from 2019 to 2021 (Table 1, Supplementary Material 1). The continuous variation observed in GNS in the RIL population suggests that it is a quantitatively inherited trait with typical characteristics, making it suitable for QTL analysis (Fig. 1B-G).

**Table 1:** Comparative analysis of GNS in Pubing3228/Jing4839 RIL population across different environments

Environment	Years	Parents		GWP		RIL population						
		Pubing3228	Jing4839	Pubing3228	Jing4839	Maximum	Minimum	Mean ± SD	Range	CV(%)	Skewness	Kurtosis
**E**_**1**_	2019	88	32.5	6.44	3.09	95.67	29.5	55.28 ± 12.63	66.17	22.84	0.79	0.62
**E**_**1**_	2020	87.25	24	6.39	2.28	113	28	59.69 ± 14.76	85	24.73	0.85	1.39
**E**_**2**_	2020	91.15	26.28	6.68	2.5	84.67	25	53.78 ± 10.30	59.67	19.16	0.28	0.29
**E**_**3**_	2020	89.34	29.61	6.54	2.81	97	24	58.13 ± 13.09	73	22.53	0.35	-0.08
**E**_**1**_	2021	92.31	36.07	6.76	3.43	140	22	73.75 ± 22.79	118	31.72	0.57	0.12
**E**_**2**_	2021	90.06	25.18	6.59	2.4	112	19.67	47.81 ± 12.72	92.33	26.61	0.88	2.59

*Note* E1, E2, E3 represent environments at Pingdingshan, Yangling, and Xianyang respectively. RIL: Recombinant Inbred Line; SD: Standard Deviation; CV: Coefficient of Variation. Significance levels are indicated with the symbols ‘a’ and ‘b’

Notably, the highest GNS, approximately 140 grains, was observed in the Pingdingshan (E1) environment in 2021, while the lowest GNS, about 19.67 grains, was recorded in the Yangling (E2) environment, indicating significant environmental effects on GNS (Table 1). Moreover, the average GNS of the RIL population ranged from 47.81 to 59.69 grains across different environments, implying an interaction between genetic factors and environmental conditions in influencing GNS (Table 1). The near-normal distribution of GNS data, with skewness and kurtosis absolute values mostly less than 1, further supported the suitability of this trait for quantitative genetic analysis (Table 1). Significant differences in GNS were observed between Pubing3228 and Jing4839, as well as variations in the RIL population under different environments.

### Analysis of the genetic structure of hexaploid wheat through high-density linkage mapping

In this study, we constructed a high-density genetic linkage map using the genetic diversity analysis of two wheat varieties, Pubing3228 and Jing4839. Our approach involved using a 55 K iSelect SNP array, through which we selected 3,334 polymorphic markers out of a total of 52,478 SNP markers. These markers, which exhibited differences between the two varieties, were used to build the map (Table 2). To ensure the map’s accuracy and reliability, we excluded markers missing more than 10% of individuals or exhibiting co-segregation at the exact location.

**Table 2:** High-density genetic linkage maps of Pubing3228/Jing4839 RIL population

Chromosome	Primary SNPs	Loci	Length(cM)	Max spacing(cM)	Min spacing(cM)	Ave. spacing(cM)
1A	2640	190	168.99	8.61	0.2439	0.89
1B	2634	104	250.19	39.74	0.2427	2.41
1D	2558	144	586.92	47.64	0.2427	4.08
2A	2634	123	217.25	30.09	0.2451	1.77
2B	2646	128	232.97	40.99	0.2415	1.82
2D	2649	172	684.04	45.56	0.2404	3.98
3A	2208	217	362.14	35.05	0.2415	1.67
3B	2647	154	225.22	18.19	0.2451	1.46
3D	2111	160	709.68	39.76	0.2415	4.44
4A	2639	145	184.83	16.35	0.2439	1.27
4B	2638	133	154.07	18.08	0.2404	1.16
4D	1613	76	685.98	46.67	0.2439	9.03
5A	2653	165	497.04	49.94	0.2415	3.01
5B	2658	187	283.98	12.77	0.2415	1.52
5D	2200	211	1060.59	52.76	0.2415	5.03
6A	2649	108	160.39	17.83	0.2415	1.49
6B	2667	125	295.18	47.89	0.2404	2.36
6D	2112	126	498.13	39.02	0.2427	3.95
7A	2654	218	486.81	41.49	0.2392	2.23
7B	2596	186	429.73	46.41	0.2392	2.31
7D	2653	262	1320.22	45.66	0.2415	5.04
Genome A	18077	1166	2077.44	49.94	0.2392	1.76
Genome B	18486	1017	1871.35	47.89	0.2392	1.86
Genome D	15896	1151	5545.57	52.76	0.2404	5.08
Overall	52459	3334	9494.36	52.76	0.2392	2.90

*Note* SNP: single nucleotide polymorphism; cM: centiMorgans

The constructed linkage map accurately represented each chromosome of hexaploid wheat, resulting in 21 linkage groups. Notably, 16 chromosomes were selected for this study (Supplementary Material 2). The total map length was 9,494.36 cM, with an average interval of 2.90 cM between adjacent markers. Notably, the number of markers and marker intervals varied across specific chromosomes. For example, there were 76 markers on chromosome 4D, while chromosome 7D had 262 markers. The most minor average interval, 0.89 cM, was observed on chromosome 1 A, whereas the most significant average interval, 9.03 cM, was found on chromosome 4D.

Regarding the genome breakdown, the linkage map for the A genome comprised 1,166 markers, accounting for 34.97% of the total markers. It had a total length of 2,077.44 cm, with an average interval of 1.76 cm. The B genome consisted of 1,017 markers, representing 30.50% of the total markers, and had a total length of 1,871.35 cm, with an average interval of 1.86 cm. On the other hand, the D genome contained 1,151 markers, accounting for 34.53% of the total markers. It had a total length of 5,545.57 cm, with an average interval of 5.08 cm. Interestingly, while the number of markers in the A genome was similar to that in the B and D genomes, the marker coverage in the B genome was lower, suggesting potential differences in genetic diversity.

### Analysis of QTLs for spikelet number revised to grain number per spike in wheat across multiple environments

GNS is a crucial trait that determines wheat yield. Understanding the genetic basis of GNS is vital for breeding. Thus, we conducted QTL mapping of GNS in two populations, Pubing3228 and Jing4839, under different environmental conditions to identify the key genetic factors controlling GNS.

Between 2019 and 2021, across three diverse environments, a total of 40 QTLs related to GNS were identified, distributed across 16 chromosomes, encompassing all chromosomes except for 1B, 2A, 5B, 6A, and 7A (Table 3). Chromosomes 5D and 7D harbored the highest number of QTLs, with six identified on each, whereas chromosomes 2B, 3B, 4D, 6B, and 6D each had only one QTL identified. The log of odds (LOD) scores of these QTLs ranged from 3.01 to 33.21, accounting for 4.25–17.17% of the phenotypic variation. Among all detected QTLs, Q*Gns.wa-1 A.e1.2* exhibited the highest percentage of phenotypic variation explained (PVE), reaching up to 17.17%, located between AX-94,465,571 and AX-109,859,174 on chromosome 1 A. Following this were Q*Gns.wa*-*7Da.e1* and Q*Gns.wa*-*1D.e1.1*, contributing 15.12% and 14.83% of PVE, respectively. Four QTLs were consistently detected across different environments, indicating stability, including Q*Gns.wa*-*1A.e1.2*, Q*Gns.wa*-*1D.e1.1*, Q*Gns.wa*-*5A.e1*, and Q*Gns.wa*-*7Da.e1*, all showing high PVE. Q*Gns.wa*-*1A.e1.2* on chromosome 1A was detected in environments 2021E1, 2020E2, and 2020E3, contributing 8.2–17.17% to phenotypic variation. Q*Gns.wa*-*1D* on chromosome 1D was observed in environments 2019E1, 2020E1, and 2020E3, contributing 9.89–14.83% to phenotypic variation. Q*Gns.wa*-*5A* on chromosome 5A was identified in environments 2020E1, 2020E2, and 2020E3, explaining 9.095–11.13% of the phenotypic variation. Q*Gns.wa*-*7Da.e1* on chromosome 7D accounted for 4.92–15.12% of the phenotypic variation. Notably, positive additive effects indicated an increase in the effect from Pubing3228, while negative additive effects suggested an increase from Jing4839. Except for two QTLs, all others exhibited positive additive effects, indicating that all favorable alleles originated from Pubing3228, which carries genetic traits promoting high GNS.

**Table 3:** Significant QTLs identified for GNS in the Pubing3228/Jing4839 RIL populations across different environments

Environment	Years	QTL^a^	Marker interval	Position(cM)	LOD	PVE(%)	A^b^
E_1_	2019	QGns.wa-4B.e1	AX-109526283–AX-109469073	15	4.54	8.63	3.63
E_1_	2019	QGns.wa-5A.e1.1	AX-109285497–AX-110430928	164	3.3	6.49	3.15
E_1_	2019	QGns.wa-5A.e1.2	AX-109959220–AX-108905072	334	6.39	12.75	4.36
E_1_	2019	QGns.wa-5D.e1.1	AX-110478685–AX-109292429	409	3.77	10.61	4.83
E_1_	2019	QGns.wa-5D.e1.2	AX-89633041–AX-89700472	809	3.37	4.92	3.27
E_1_	2019	QGns.wa-6B.e1	AX-111038900–AX-111601539	188	3.23	7.31	3.16
E_1_	2019	QGns.wa-6D.e1	AX-108805031-AX-111480830	244	3.99	9.17	3.54
E_1_	2019	QGns.wa-7B.e1.1	AX-94770114-AX-110935237	50	4.16	4.69	3.13
E_1_	2019	QGns.wa-7B.e1.2	AX-111171468–AX-111044632	422	3.56	5.73	3.47
E_1_	2019	QGns.wa-7Da.e1.2	AX-111061288–AX-110826147	106	9.4	11.21	4.85
E_1_	2020	QGns.wa-1A.e1.2	AX-110669146–AX-110584864	168	4.17	10.25	4.21
E_1_	2020	QGns.wa-7Da.e1.1	AX-108759691–AX-110520245	64	3.45	5.45	-3.87
E_1_	2021	QGns.wa-1D.e1	AX-94777221–AX-108768444	126	3.05	7.94	6.14
E_1_	2021	QGns.wa-2B.e1	AX-110932072–AX-111722030	232	3.52	8.92	6.64
E_1_	2021	QGns.wa-2D.e1	AX-109913269–AX-111678720	362	3.09	7.69	6.02
E_1_	2021	QGns.wa-5A.e1.1	AX-109861283–AX-109931609	265	3.77	7.2	6.77
E_1_	2021	QGns.wa-5D.e1	AX-110986471–AX-94458300	519	4.01	10.05	7.25
E_1_	2019, 2020	QGns.wa-3D.e1	AX-111579109–AX-111676471	576	3.04–4.39	8.76–9.96	4.71–6.54
E_1_, E_1_, E_3_	2020, 2019, 2020	QGns.wa-1D.e1.1	AX-109994213–AX-89491149	128	4.41–7.36	9.89–14.83	4.19–5.12
E_1_, E_2_	2020	QGns.wa-5D.e1	AX-108803037–AX-110591185	835	3.15–3.99	6.67–8.14	3.32–4.17
E_1,_ E_2_, E_3_	2020	QGns.wa-5A.e1	AX-108905072–AX-109108075	338	4.7–5.48	9.09–11.13	3.17–5.16
E_1_, E_2_, E_3_	2021, 2020, 2020	QGns.wa-1A.e1.2	AX-94465571–AX-109859174	167	3.01–8.39	8.2-17.17	3.58–6.08
E_1_, E_2_, E_3_	2021, 2020, 2020	QGns.wa-7Da.e1	AX-110826147–AX-108748734	110	3-11.42	4.92–15.12	2.7–7.97
E_1_, E_3_	2019, 2020	QGns.wa-4A.e1	AX-108908317–AX-109987309	131	3.36–3.44	4.6–6.28	3.21–3.44
E_1_, E_3_	2019, 2020	QGns.wa-7Da.e1.3	AX-111638626–AX-109833182	343	4.55–4.78	12.86–13.82	5.19–5.38
E_2_	2020	QGns.wa-1D.e2	AX-108768444–AX-109994213	127	5.84	11.71	3.5
E_2_	2020	QGns.wa-2D.e2	AX-110412287–AX-110574726	525	3.18	7.08	2.91
E_2_	2020	QGns.wa-3B.e2	AX-110525210–AX-110931375	51	3.08	7.56	2.58
E_2_	2020	QGns.wa-4D.e2	AX-111002463–AX-89617545	83	3.24	6.76	2.73
E_2_	2020	QGns.wa-7B.e2	AX-108795893–AX-109871179	56	3.65	5.45	2.74
E_2_	2020	QGns.wa-7Da.e2.2	AX-110538984–AX-110625335	415	4.76	9.81	3.68
E_3_	2020	QGns.wa-3A.e3.1	AX-109478387–AX-109956153	203	33.21	13.61	-12.02
E_3_	2020	QGns.wa-3A.e3.2	AX-111451084–AX-111531488	208	23.89	8.74	9.84
E_3_	2020	QGns.wa-3D.e3	AX-110525165–AX-109416738	433	4.65	8.59	3.76
E_3_	2020	QGns.wa-4A.e3.2	AX-108877111–AX-109936672	184	3.72	6.85	3.36
E_3_	2020	QGns.wa-4B.e3	AX-109385774–AX-112287589	32	4.9	9.69	3.73
E_3_	2020	QGns.wa-5A.e3.2	AX-109369427–AX-110020985	146	3.69	7.19	3.26
E_3_	2020	QGns.wa-5D.e3.1	AX-109510714–AX-94468261	5	3.99	7.98	3.91
E_3_	2020	QGns.wa-5D.e3.2	AX-110421468–AX-108803037	826	4.95	9.91	4.35
E_3_	2020	QGns.wa-7Db.e3.5	AX-110504662–AX-111707163	379	3.53	4.25	2.99

*Note* E1, E2, E3 refer to Pingdingshan,: Yangling,:Xianyang, respectively. ^a^Nomenclature for QTL in wheat: ‘‘Q’’ refers to QTL, followed by a trait designator, ‘‘wa’’ for the laboratory, and chromosome. ^b^Positive additive effects indicate increased effects from Pubing3228, and negative additive effects indicate increased effects from Jing 4839

### Exploration of the molecular mechanisms of GNS in wheat

To clarify the molecular mechanism behind GNS in wheat, this study analyzes stable QTLs that impact GNS. The study constructs an expression heatmap of relevant genes using the Wheat Expression Browser public database (http://www.wheatexpression.com). Out of the 1265 genes analyzed, emphasis is placed on genes expressed explicitly in wheat grains, considering them as candidate genes that may influence GNS. This approach identifies 11 potential candidate genes (Table 4) related to GNS. These genes are primarily located on chromosomes 4A, 5D, and 7D.

**Table 4:** Identified candidate genes for GNS in wheat from the Pubing3228/Jing4839 RIL population

No^a^	Chromosome	Identified loci in current study	Position (bp)^b^	Candidate genes (closest/nearby)	Annotation
1	4A	AX-108908317	679194458	TraesCS4A01G601800	4-hydroxybenzoate octaprenyltransferase
2	5D	AX-108803037	27452078	TraesCS5D01G044000	60S ribosomal protein l28
3	5D	AX-108803037	31475580	TraesCS5D01G051000	Senescence-associated protein
4	5D	AX-110591185	32303148	TraesCS5D01G033700	YABBY transcription factor
5	7D	AX-108759691	29882930	TraesCS7D01G056100	S-adenosylmethionine decarboxylase proenzyme
6	7D	AX-108759691	32673051	TraesCS7D01G060100	Cysteine protease
7	7D	AX-110520245	35599225	TraesCS7D01G064300	Starch synthase
8	7D	AX-110826147	65750064	TraesCS7D01G109200	GDSL esterase/lipase
9	7D	AX-108748734	74057072	TraesCS7D01G148000	Senescence-associated protein
10	7D	AX-108748734	72522918	TraesCS7D01G117600	Ethylene-responsive transcription factor
11	7D	AX-108748734	72216910	TraesCS7D01G117100	Histone H3

*Note*^a^The number of candidate genes for wheat grain Fe concentration. ^b^Physical position of the SNP as reported in the IWGSC Chinese Spring reference genome RefSeq v2.0

Among the candidate genes, *TraesCS4A01G601800* is the only one on chromosome 4A. It is potentially involved in the function of 4-hydroxybenzoate acetyltransferase in wheat. On chromosome 5D, three candidate genes are identified: *TraesCS5D01G044000*, *TraesCS5D01G051000*, and *TraesCS5D01G033700*. These genes may encode ribosomal protein L28 [49], aging-related protein, and YABBY transcription factor. On chromosome 7D, seven candidate genes with diverse functions are found. They include *TraesCS7D01G056100*, potentially encoding S-adenosylmethionine decarboxylase precursor, *TraesCS7D01G060100* for cysteine proteinase, *TraesCS7D01G064300* for starch synthase, *TraesCS7D01G109200* for GDSL esterase/lipase, *TraesCS7D01G148000* for an aging-related protein, *TraesCS7D01G117600* for an ethylene-responsive transcription factor, and *TraesCS7D01G117100* for histone H3 [50, 51].

## Discussion

GNS, a key determinant of wheat yield, has been extensively studied [52]. Previous research has attributed the enhancement of wheat yield primarily to the increase in GNS [53], making it a crucial selection target in wheat breeding practices. Despite significant progress in GNS over the past decades in China, which has substantially contributed to yield improvements in wheat breeding, recent years have not seen notable advancements in wheat yield enhancement. This stagnation may be attributed to slow progress in identifying and utilizing new genetic resources related to GNS [54, 55]. The germplasm Pubing3228, derived from distant hybridization between common wheat and Agropyron, represents a novel wheat germplasm with numerous desirable agronomic traits, especially those associated with GNS. Therefore, it is imperative to further explore the genetic mechanisms underlying the high grain number in Pubing3228 [56, 57].

### QTL comparative study of genetic mechanisms for GNS in Pubing3228 wheat germplasm

We conducted a study using an F_2:3_ population derived from a cross between Pubing3228 and Jing4839 to investigate the underlying genetic mechanism of GNS in Pubing3228. Our investigation revealed 12 QTLs spread across multiple chromosomes (Supplementary Material 3), marking a notable increase to 40 identified QTLs, with many not previously detected. This variance could stem from using an RIL population and a higher-density genetic linkage map constructed with 3334 SNP markers, unlike the earlier study’s temporary F_2:3_ population and 179 SSR markers.

We employed the BLAST method to determine the physical positions of the QTL identified in the previous study. It is important to note that the QTLs identified in our study were found within or near the mapping intervals of the previous QTL (Supplementary Material 3). For example, a previously identified QTL on chromosome 5A has been refined in our study from a 20 Mb interval, highlighting potential consistency between the two studies and narrowing the mapping intervals for several QTLs, such as those on chromosomes 7A and 7B. This detailed comparison underscores the consistency of our findings with previous research while offering more precise mapping intervals for identified QTLs.

### Comparative analysis and new insights of QTLs for GNS in Pubing3228 wheat germplasm

In this study, we delved into the genetic underpinnings of GNS to enhance wheat yield and facilitate MAS in breeding. Through QTL analysis, an established method for uncovering genetic resources, we reviewed and identified 170 GNS QTLs across all 16 chromosomes (Supplementary Material 4) [58, 59], aligning the physical positions of 153 previously reported QTLs for comparison (Supplementary Material 4). Comparing our results with previous studies revealed some significant differences. However, our study reveals that the genomic locations of several QTLs, which account for approximately 21.33% of the total variation, are consistent with previously identified QTLs (Supplementary Material 5). Our findings showed significant differences and consistencies with prior research, notably confirming the locations of several QTLs that contribute to about 21.33% of the total variation. For instance, we identified QTLs on chromosome 1 A at the same position (498–499 Mb) as previously reported and discovered three GNS QTLs within a broad genetic region on chromosome 7B, previously noted in another study [60, 61]. Our research also matched previously identified GNS QTLs on chromosomes 5 A and 6B [62].

In this study, we identified several GNS QTLs that were consistent with previous research, indicating the reliability of our results. We mapped three major QTLs (Q*Gns.wa-1A*, Q*Gns.wa-7D*, and Q*Gns.wa-1D*) to specific regions on chromosomes 1A, 7D, and 1D, respectively. These regions were between AX-94,465,571 and AX-109,859,174, AX-110,826,147 and AX-108,748,734, AX-109,994,213 and AX-89,491,149. Among the QTLs identified, these QTLs exhibit the highest PVE in all three environments and demonstrate stability. However, the other two major GNS QTLs have yet to be documented in previous studies [63, 64].

An exciting aspect of our study is that we detected more GNS QTLs than in previous studies. This condition may be attributed to the utilization of new germplasm Pubing3228, which is derived from a cross between common wheat and *A. cristatum*. Moreover, to enhance the detection of GNS QTLs, we constructed a high-density linkage map using many gene-based SNP markers. It is important to note that our study did not find GNS QTLs on chromosomes 5B or 6A. However, previous studies have identified these QTLs controlling GNS [60, 65].

### Essential chromosomes and region analysis of QTL for GNS in Pubing3228 wheat germplasm

This study observed an uneven distribution of QTLs for GNS across different chromosomes. Most GNS QTLs were concentrated on a small subset of chromosomes, namely 1A, 1D, 3A, 3D, 5A, 5D, and 7D. For instance, chromosome 1D harbored five GNS QTLs within the range of 126 cM to 129 cM, while chromosome 5A contained five QTLs within the 326 cM to 338 cM. Although only four GNS QTLs were identified on chromosome 3A, they were concentrated in a relatively short range of 203 cM to 258 cM. Other noteworthy regions included the interval of 809 cM to 835 cM on chromosome 5D and 165 cM to 168 cM on chromosome 1A, each containing four GNS QTLs. On the other hand, GNS QTLs on chromosomes 3D and 7D were scattered throughout the chromosomes. These significant chromosomal regions should be prioritized for future research. Similar regions and chromosomes associated with GNS have been identified in previous studies [66–68]. Although various studies have explored yield-related traits in wheat, limited research has specifically investigated GNS [69–71]. A previous study identified and validated major regions on chromosomes 5A and 2A for high GNS using an RIL population derived from the hybridization between synthetic hexaploid wheat ‘W7984’ and the spring wheat variety ‘Opata M85’. Notably, two GNS QTLs were identified on chromosome 6D. Pubing3228 originated from the crossbreeding of common wheat and *A. cristatum* [70]. Prior research demonstrated that increased floret and grain numbers in the wheat-*A. cristatum* chromosome addition line 4844-12 (2n = 44) was controlled by a pair of *A. cristatum* chromosomes (6P) substituting the wheat chromosome 6D. Pubing3228 was developed through several generations of selection from the progeny of 4844-12 [72]. A prior study has also highlighted the importance of the *A. cristatum* 6P chromosome segment in increasing GNS in wheat-*A. cristatum* translocation line Pubing2978. Hence, further analysis is required to determine if these two QTLs on chromosome 6D have origins in *A. cristatum* [45].

### Prediction and analysis of candidate genes associated with GNS in Pubing3228 wheat germplasm

Although numerous QTLs associated with GNS have been identified in wheat, only some candidate genes for GNS have been reported, leaving the genetic mechanism of GNS in wheat largely unknown. This study pinpointed seven stable QTLs for GNS across various environments and proceeded with an analysis to predict 11 potential candidate genes based on functional annotation and expression levels.

Three candidate genes were particularly noteworthy due to their significant association with the most impactful SNP AX-108,748,734, on chromosome 7D, contributing to the highest average PVE (12.62%). The first, TraesCS7D01G148000, is hypothesized to encode a protein linked to aging processes in wheat, crucial for grain yield as they potentially facilitate nutrient reallocation to developing seeds [73]. The second, TraesCS7D01G117600, is believed to encode an ethylene-responsive transcription factor (ERF), part of the AP2/ERF family, instrumental in stress response, reproduction, defense, and hormone secretion regulation [74], with implications for flower development control [75]. The third, TraesCS7D01G117100, might be associated with histone H3, playing a role in the vernalization-induced transition from vegetative to reproductive growth through histone methylation [50, 51], suggesting its potential impact on GNS due to the established relationship between GNS and growth phases [76].

Four other candidate genes were also identified on chromosome 7D. Two genes (*TraesCS7D01G056100*, *TraesCS7D01G060100*) correspond to SNP AX-108,759,691, with *TraesCS7D01G056100* speculated to encode S-adenosylmethionine decarboxylase precursor, potentially playing a role in polyamine biosynthesis. Research has suggested that polyamines, including putrescine, spermidine, and cadaverine, are involved in cell division, embryogenesis, flower development, and fruit development [77, 78]. The candidate gene *TraesCS7D01G060100* may encode a cysteine protease, a significant class of proteases in plants involved in various processes such as post-translational modification, development, aging, programmed cell death, and antibiotic response [79, 80]. The candidate genes *TraesCS7D01G064300* and *TraesCS7D01G109200* may encode starch synthase and GDSL esterase/lipase, respectively. Starch synthase is a critical enzyme in starch synthesis in wheat endosperm, directly affecting final yield [81, 82], while GDSL esterase/lipase plays a regulatory role in plant development and morphogenesis [83, 84].

On chromosome 5D, three candidate genes have been pinpointed: *TraesCS5D01G044000*, *TraesCS5D01G051000*, and *TraesCS5D01G033700*. *TraesCS5D01G044000* and *TraesCS5D01G051000* are postulated to encode for the 60 S ribosomal protein l28 and a protein linked to aging processes, respectively. The 60 S ribosomal protein l28 plays a crucial role in translation, potentially influencing mitochondrial translation and various plant cellular activities [85]. Research indicates that the 60 S ribosomal proteins are integral to maintaining ribosomal complex stability and enhancing protein biosynthesis, suggesting their possible impact on GNS. Additionally, *TraesCS5D01G033700* is implicated in the regulation of pistil and stamen development within wheat flowers. Various studies have documented the significant expression of TaYABBY genes throughout cereal development phases, underscoring their importance in plant growth and reproduction [86, 87]. Furthermore, a significant SNP, AX-108,908,317, on chromosome 4A has been associated with a gene encoding 4-hydroxybenzoic acid acetyltransferase. Previous research has demonstrated that this enzyme is crucial for ubiquinone biosynthesis in rice [88]. Ubiquinone is an essential lipophilic electron carrier required for the mitochondrial respiratory chain in eukaryotic cells [89, 90]. In *Arabidopsis*, studies have shown that mutants lacking ubiquinone experience developmental arrest during early embryogenesis stages [91–94].

### Scientific and production significance

This study offers significant scientific value in providing new avenues and tools for enhancing the genetic potential of wheat. Firstly, by analyzing the hybridization of Pubing3228 and Jing4839 and conducting subsequent QTL analysis, this study not only elucidates the genetic basis of GNS but also identifies valuable molecular markers that can be employed in wheat breeding programs. Developing high-yielding wheat varieties can be expedited by utilizing these markers in MAS. The identification and analysis of GNS-related genes in this study contribute to our understanding of the underlying mechanisms involved in wheat yield formation, thereby offering crucial insights for future efforts in crop improvement. Moreover, although this study primarily focuses on plant genetics, its methodology and findings extend beyond this area and can inspire a diverse range of biological and agricultural research. For instance, comprehending the genetic control mechanisms behind complex traits like GNS can facilitate similar investigations in other crops or biological traits.

### Limitations and future prospects

Despite the advancements achieved in our research, certain limitations require attention. While candidate genes for GNS have been pinpointed, their exact roles and mechanisms in GNS regulation need experimental validation for a comprehensive understanding. Additionally, the impact of environmental variables on GNS was not exhaustively analyzed. Future studies should evaluate the stability and performance of QTLs across various environmental settings. This investigation predominantly centered on the Pubing3228 wheat germplasm, potentially restricting the universality of the identified QTLs and genes. Expanding research to include a broader spectrum of wheat germplasms will broaden the relevance and application of these findings. Employing precise gene-editing methods, such as CRISPR/Cas9, to alter candidate genes can validate their functions and investigate their suitability for molecular breeding strategies. In conclusion, our study contributes valuable perspectives on genetic advancement and yield optimization in wheat, yet further exploration is essential to validate these insights fully.

## Conclusion

**Fig. 2:**
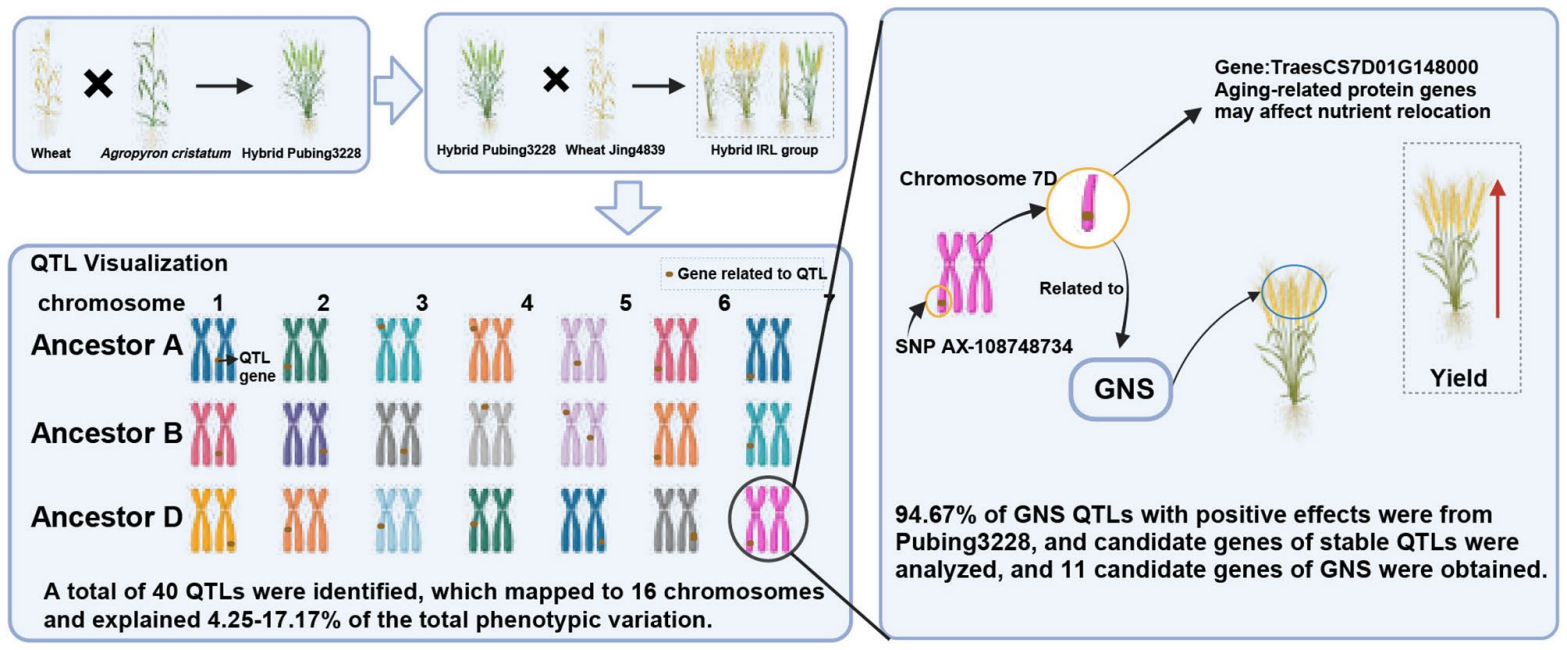
Graphical summary of the genetic basis of high GNS in the wheat-*A. cristatum* hybrid germplasm Pubing3228

This study identified 40 QTLs associated with GNS in the wheat germplasm Pubing3228 (Fig. 2), thereby enriching our understanding of wheat’s genetic diversity and illuminating the intricate genetics of the GNS trait. These QTLs, distributed across 16 chromosomes, underscore the multi-gene influence on GNS rather than its being governed by a single gene. Five QTLs demonstrated substantial phenotypic variability across different environments, accounting for over 10% of the variation and highlighting their importance in wheat yield development. The majority (approximately 94.67%) of beneficial GNS QTLs were derived from Pubing3228, underscoring its genetic resource value. Furthermore, the study proposes 11 candidate genes potentially influencing GNS, involving processes like aging and ethylene response, offering crucial insights into the genetic regulatory mechanisms of GNS.

### Electronic supplementary material

Below is the link to the electronic supplementary material.

Supplementary Material 1

Supplementary Material 2

Supplementary Material 3

Supplementary Material 4

Supplementary Material 5

## Data Availability

All data generated or analysed during this study are included in this published article.

## Abbreviations

GNS: Grain number per spike
RIL: Recombination inbred line
SNP: Single nucleotide polymorphism
QTL: Quantitative trait locus
MAS: Marker-assisted selection
NGS: Next-generation sequencing
MTA: Marker-trait association
GISH: Genomic in situ hybridization
FISH: Fluorescence in situ hybridization
PDS: Pingdingshan
YL: Yangling
XY: Xianyang
PVE: Phenotypic variance explanation
