# Supplementary material for: Genetic insights into superior grain number traits: a QTL analysis of wheat-Agropyron cristatum derivative pubing3228
Source: BMC Plant Biol. 2024 Apr 11;24:271. doi: 10.1186/s12870-024-04913-z (PMC11008026; doi:10.1186/s12870-024-04913-z)
Supplement: Supplementary file 5 — Supplementary Material 5 [file 12870_2024_4913_MOESM5_ESM.docx]

1A 1B

**Supplementary Material 2. Comprehensive Genetic Maps Illustrating QTL Associated with GNS in the Pubing3228/Jing4839 RIL Population**


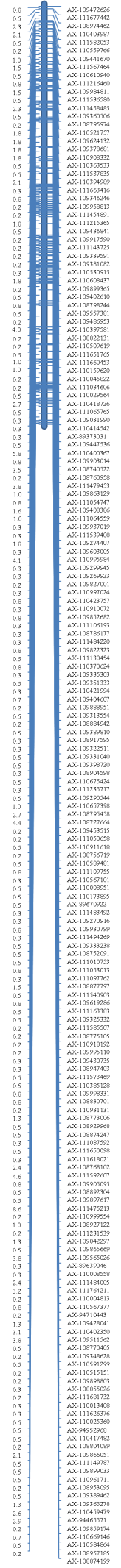

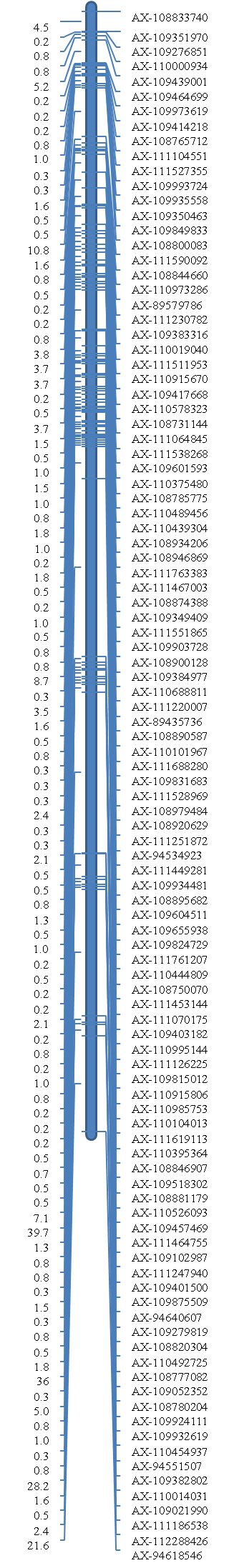


QGns.wa-1A.e1.1（2020）

QGns.wa-1B.e2（2020）

QGns.wa-1A.e1.1（2021）

QGns.wa-1A.e3（2020）

QGns.wa-1A.e2（2020）

QGns.wa-1A.e1.2（2021）

QGns.wa-1A.e1.2（2020）

1D 2A


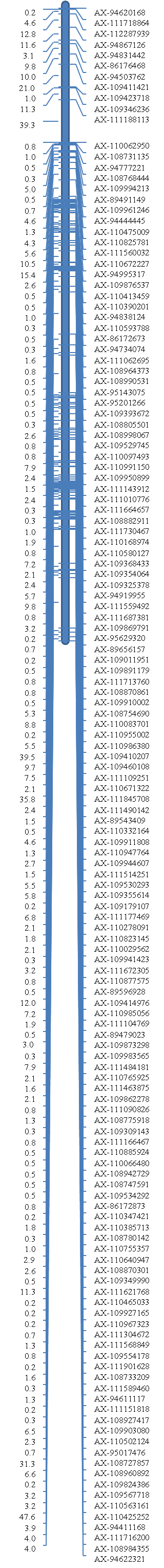

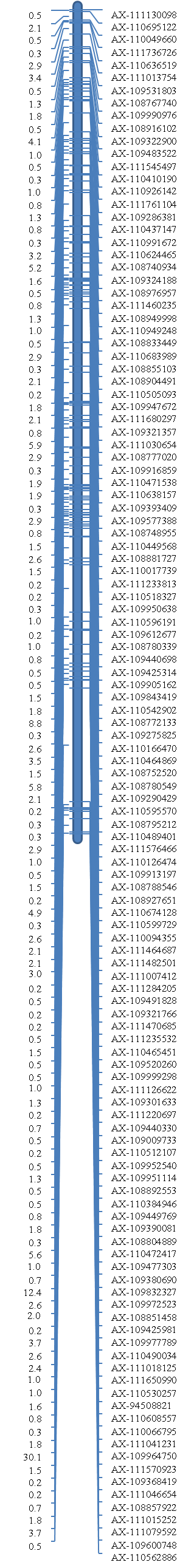


QGns.wa-1D.e3（2020）

QGns.wa-1D.e1.1（2019）

QGns.wa-1D.e1（2021）

QGns.wa-1D.e2（2020）

QGns.wa-1D.e1.1（2020）

QGns.wa-1D.e1.2（2020）

QGns.wa-1D.e3（2020）

QGns.wa-1D.e1.2（2019）

QGns.wa-2A.e1（2021）

Fig.2 (Continued)

2B 2D


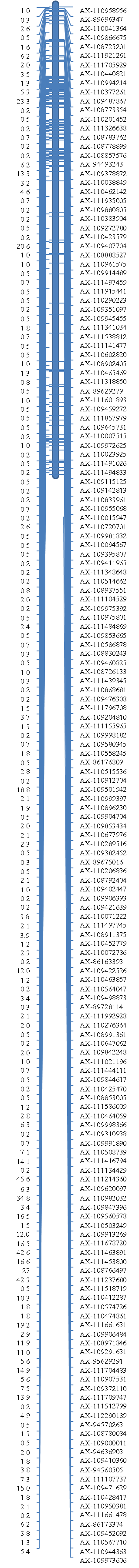

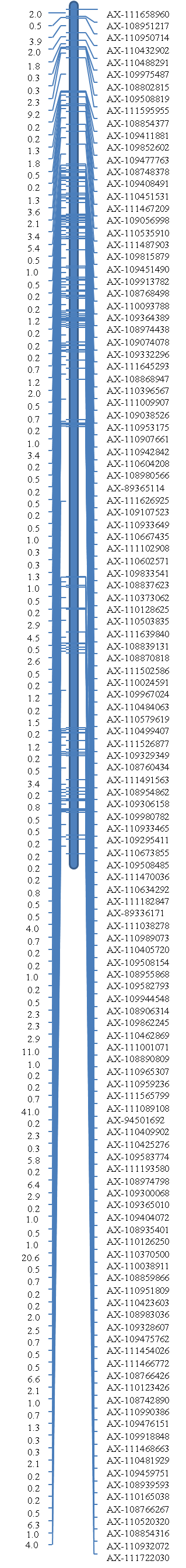


QGns.wa-2B.e2（2020）

QGns.wa-2B.e1（2019）

QGns.wa-2D.e1（2021）

QGns.wa-2D.e2（2020）

QGns.wa-2B.e1（2021）

Fig.2 (Continued)

3A 3B


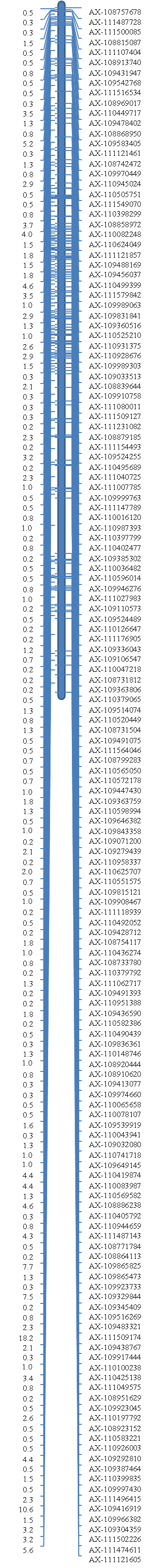

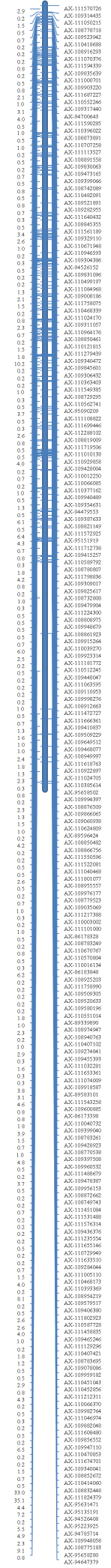


QGns.wa-3B.e2（2020）

QGns.wa-3A.e3.1（2020）

QGns.wa-3A.e3.2（2020）

QGns.wa-3A.e1（2021）

QGns.wa-3A.e1（2020）

Fig.2 (Continued)

3D 4A


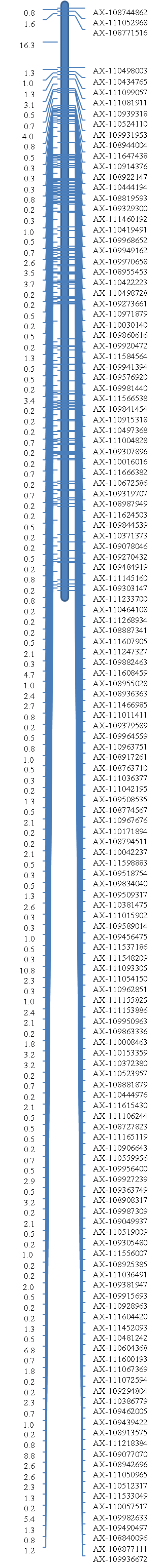

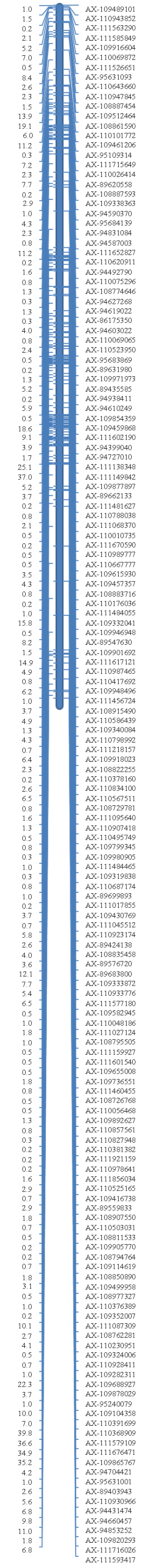


QGns.wa-3D.e1（2021）

QGns.wa-3D.e3（2020）

QGns.wa-4A.e3.1（2020）

QGns.wa-4A.e1（2019）

QGns.wa-3D.e2（2020）

QGns.wa-4A.e3.2（2020）

QGns.wa-3D.e1（2019）

QGns.wa-3D.e1（2020）

Fig.2 (Continued)

4B 4D


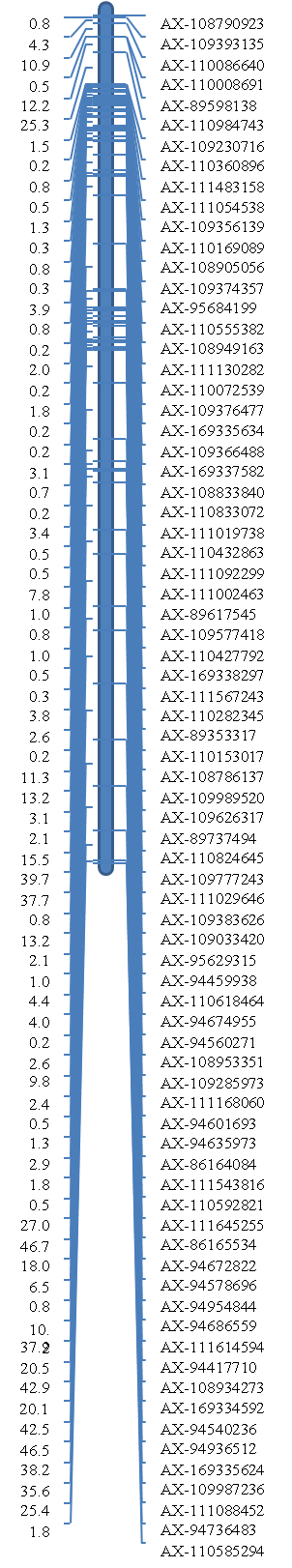

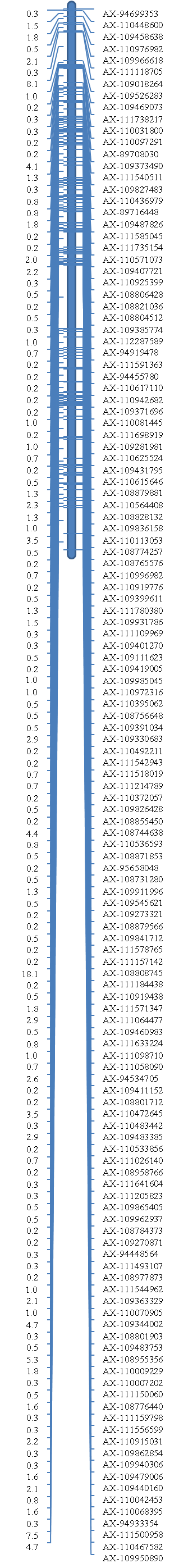


QGns.wa-4B.e1（2019）

QGns.wa-4B.e3（2020）

QGns.wa-4D.e2（2020）

Fig.2 (Continued)

5A 5D

QGns.wa-5D.e3.1（2020）


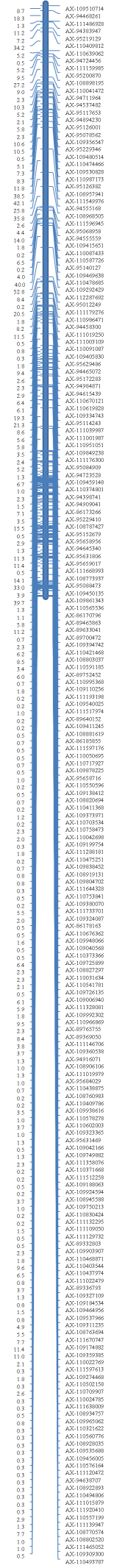

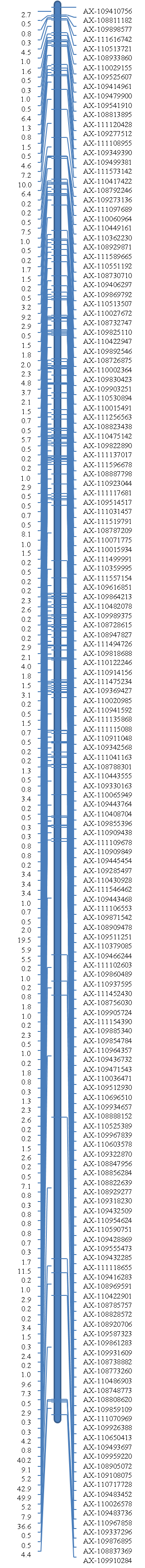


QGns.wa-5A.e3.1（2020）

QGns.wa-5D.e1.1（2019）

QGns.wa-5D.e1（2021）

QGns.wa-5D.e1.2（2019）

QGns.wa-5A.e3.2（2020）

QGns.wa-5D.e2（2020）

QGns.wa-5D.e3.2（20020）

QGns.wa-5D.e1（2020）

QGns.wa-5A.e1.1（2019）

QGns.wa-5A.e1.1（2021）

QGns.wa-5A.e1（2020）

QGns.wa-5A.e1.2（2019）

QGns.wa-5A.e3.3（2020）

QGns.wa-5A.e2（2020）

QGns.wa-5A.e1.2（2021）

Fig.2 (Continued)

6B 6D


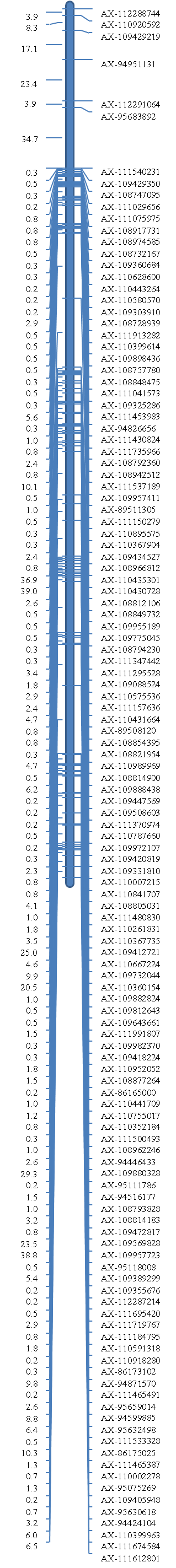

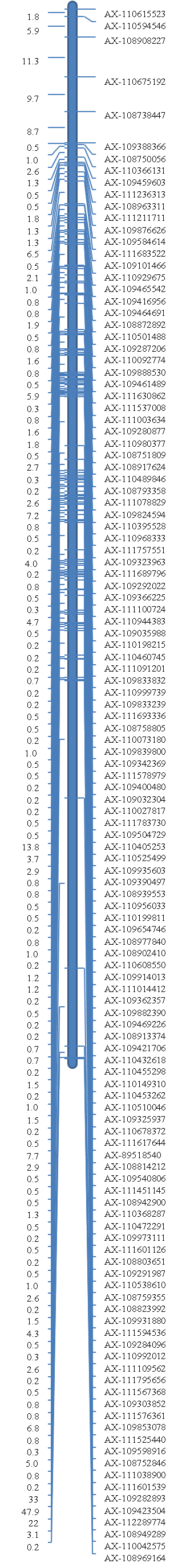


QGns.wa-6D.e3（2020）

QGns.wa-6D.e1（2019）

QGns.wa-6B.e1（2020）

QGns.wa-6B.e1（2019）

Fig.2 (Continued)

7A 7B


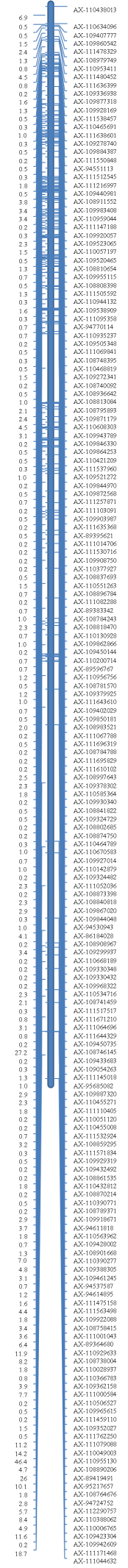

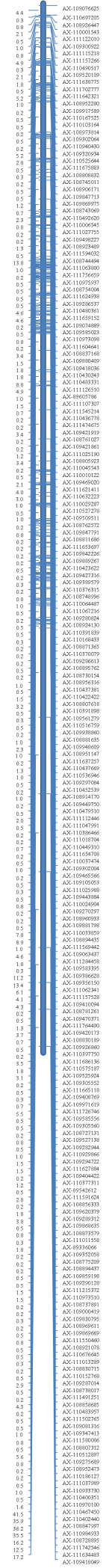


QGns.wa-7B.e1.1（2019）

QGns.wa-7B.e2（2020）

QGns.wa-7A.e3（2020）

QGns.wa-7B.e1.2（2019）

Fig.2 (Continued)

7Da 7Db


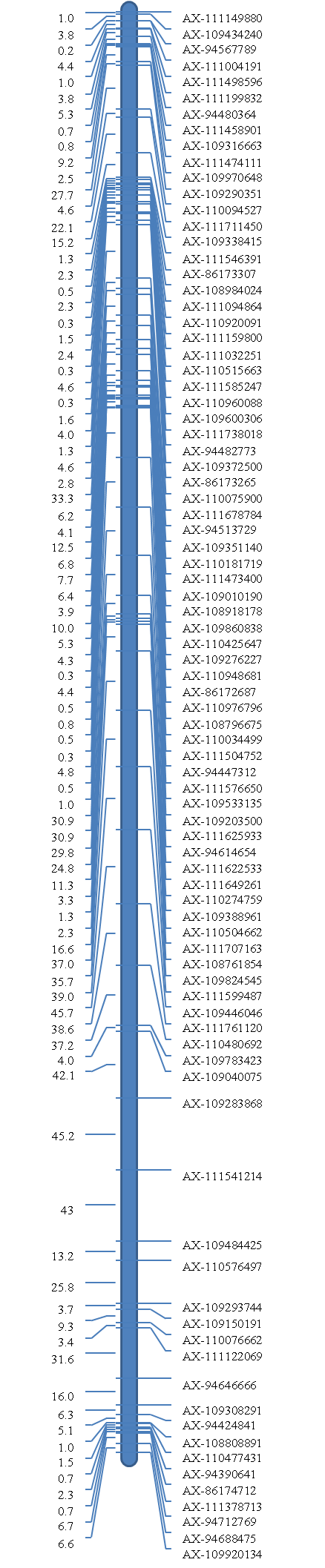

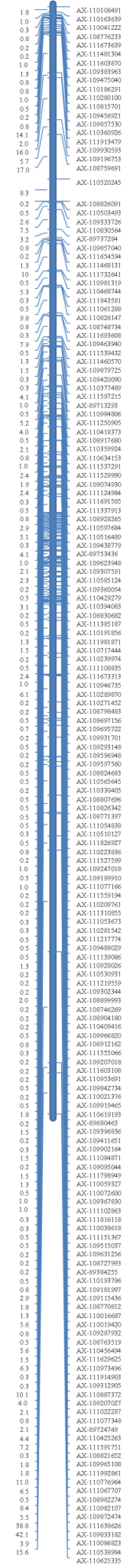


QGns.wa-7Da.e1.1（2020）

QGns.wa-7Da.e3.1（2020）

QGns.wa-7Da.e1.1（2019）

QGns.wa-7Da.e12（2019）

QGns.wa-7Da.e2.1（2020）

QGns.wa-7Da.e1（2021）

QGns.wa-7Da.e1.2（2020）

QGns.wa-7Da.e3.2（2020）

QGns.wa-1Db.e3（2020）

QGns.wa-7Da.e1.3（2020）

QGns.wa-7Da.e3.3（2019）

QGns.wa-7Da.e1.3（2019）

QGns.wa-7Da.e3.4（2019）

QGns.wa-7Da.e2.2（2020）

Fig.2 (Continued)
